# COVID-19 in-hospital mortality during the first two pandemic waves, at Helen Joseph Hospital, South Africa

**DOI:** 10.11604/pamj.2023.45.5.39222

**Published:** 2023-05-03

**Authors:** Joanna Reid, Reyna Daya, Zvifadzo Matsena Zingoni, Waasila Jassat, Zaheer Bayat, Jeremy Nel

**Affiliations:** 1 Department of Internal Medicine, Helen Joseph Hospital, Faculty of Health Sciences, University of the Witwatersrand, Johannesburg, South Africa,; 2 Division of Epidemiology and Biostatistics, Faculty of Health Sciences, University of the Witwatersrand, Johannesburg, South Africa,; 3 National Institute for Communicable Diseases (NICD), National Health Laboratory Service (NHLS), South Africa,; 4 Right to Care, Centurion, South Africa

**Keywords:** COVID-19, SARS-CoV-2, hospital mortality, risk factors

## Abstract

**Introduction:**

there has been significant global variation in Coronavirus Disease (COVID-19) mortality at different time points in the pandemic. Contributing factors include population demographics, comorbidities, health system capacity, prior infection with COVID-19, vaccinations, and viral variants. The study aims to describe COVID-19-related mortality of inpatients at Helen Joseph Hospital (HJH), over 12 months, during the first two waves of the COVID-19 pandemic in South Africa. The primary objectives were to describe the socio-demographic details, clinical characteristics, and hospital outcomes during the first and second waves of COVID-19. This included an assessment of the in-hospital case fatality ratio (CFR) of patients admitted with COVID-19. The secondary objectives were to compare the socio-demographic details, clinical characteristics, and outcomes between the two waves, and to determine risk factors associated with COVID-19-related mortality.

**Methods:**

this is a retrospective cohort study of all inpatient laboratory-confirmed COVID-19 cases at HJH from 1^st^ May 2020 to 31^st^ April 2021. Data were collected by the National Institute for Communicable Diseases (NICD). Bivariate analysis was performed to describe and compare the socio-demographic characteristics, clinical characteristics, and hospital admission outcomes between the two waves. Multivariate logistic regression was used to determine risk factors for COVID-19-related mortality.

**Results:**

overall, 1359 patients were admitted, 595 in wave one, and 764 in wave two. Patients were predominantly male (52.4%), of Black African race (75.1%) with a mean age of 54.6 (standard deviation 15.4) years. The median length of stay was 8 days (interquartile range 5-14 days). In total, 73.2% (995) of patients required oxygen, 5.2% (71) of patients received mechanical ventilation, and 7.1% (96) were admitted to the high care and Intensive Care Unit (ICU). The most common comorbid illnesses were hypertension (36.7%, n=499), diabetes mellitus (26.6%, n=362), Human Immunodeficiency Virus (HIV) (10.8%, n=147), and obesity (11.0%, n=149). The in-hospital CFR during the first wave was 30.4% (181/595) and 25.5% (195/764) (p<0.001) in the second wave, and overall, in-hospital CFR was 27.7% (376/1359). The adjusted odds of death were 79% higher among patients admitted during wave one compared to wave two (aOR=1.79; 95% CI: 1.35-2.38). A one-year increase in age increased the odds of death by 4% (aOR=1.04; 95% CI: 1.03-1.05). The need for oxygen (aOR=2.17, 95%CI: 1.56-3.01) and ventilation (aOR=7.23, 95% CI: 4.02-13.01) were significant risk factors for mortality.

**Conclusion:**

prior to the availability of vaccines, COVID-19-related mortality was high and risk factors for mortality were consistent with national and international findings. This study reflects the impact of the pandemic on the South African public sector with limited resources and minimal ICU capacity.

## Introduction

Severe Acute Respiratory Syndrome Coronavirus 2 (SARS-CoV-2) is the causative agent of Coronavirus Disease 2019 (COVID-19), a severe acute respiratory illness with multiple systemic manifestations [1]. The first case of COVID-19 was reported in South Africa on 5^th^ March 2020. The COVID-19 pandemic in South Africa has been characterized by provincial heterogeneity in the timing and distribution of infections and deaths. National trends, however, have shown a clear pattern of multiple distinct waves (the period in which the national weekly incidence risk of confirmed cases increased above 30 per 100,000 individuals [2]) of infections. The peak of the first wave was in week 28 of 2020 (5-11 July), and the peak of the second wave was in the first week of 2021 (3-9 January) [3]. To date, five waves of infection have been described, and distinct viral genomic lineages/strains have been associated with each wave [4].

Risk factors associated with severe disease and mortality due to COVID-19 include demographic factors (increasing age, male sex), smoking history, obesity, and comorbidities including hypertension, diabetes mellitus (DM), cardiovascular disease, respiratory disease, chronic kidney disease, and malignancy [5-7]. There is significant variation in case fatality rates (CFR) of COVID-19 and measurements of in-hospital CFRs ranged from 12-26% globally, prior to the availability of vaccinations [7-10]. On the African continent, in-hospital CFR ranged from 4 to 40%, and studies at the time in South Africa had shown a range in CFR from 17-40% [11-14]. Risk factors associated with increased mortality in African and South African studies included demographic risk factors described globally such as increasing age and male sex, symptomatic disease, increased illness severity, and globally recognized co-morbidities described above (such as hypertension, obesity, DM etc.) [11-15]. In addition to this, Human Immunodeficiency Virus (HIV) infection and current or previous Tuberculosis (TB) infection, non-white race groups, and treatment in the public health sector were shown to be risk factors associated with COVID-19 mortality in South Africa [11]. The wide range in CFR may be due to differences in population demographics, distribution of comorbidities, testing strategies, and healthcare system capacity and resources. Other factors have changed throughout the pandemic including clinical practice guidelines, new treatment options, the impact of different genomic variants, prior COVID-19 infection, and the availability of vaccinations.

The aim of this study was to describe COVID-19-related mortality of inpatients at Helen Joseph Hospital (HJH), over 12 months, during the first two waves of the COVID-19 pandemic in South Africa, from 1^st^ May 2020 to the 30^th^ April 2021. The primary objectives were to describe the socio-demographic details, clinical characteristics, and hospital outcomes during the first and second waves of COVID-19. This included an assessment of the in-hospital CFR of patients admitted with laboratory-confirmed COVID-19. Secondary objectives were to compare the first two waves in terms of socio-demographic details, clinical characteristics, and outcomes and to determine risk factors associated with COVID-19-related mortality. This study provides a baseline description of the impact of the pandemic in the public sector, with limited resources and capacity for intensive care, prior to the arrival of vaccinations.

## Methods

**Setting:** Gauteng Province is home to a quarter of South Africa´s population (approximately 15 million people) and has had the highest total number of COVID-19 cases to date [16]. Helen Joseph Hospital (HJH) is a tertiary public hospital with 538 beds (approximately 300 medical beds) and a ten-bed Intensive Care Unit (ICU) strictly for non-COVID-19 cases. During the peak of the waves, approximately 160 beds were required for COVID-19 patients, as additional isolation wards opened according to demand. This hospital had limited ICU capacity for COVID-19-infected patients throughout the pandemic, with a 3-bed COVID-19 ICU which opened in May 2020, later increasing to 6 beds. Patients were given ventilatory support in the emergency department and in some wards, due to the limitation in ICU beds.

**Study design:** this retrospective cohort study analysed all patients admitted with laboratory-confirmed SARS-CoV-2 infection at HJH over a year-long period (1^st^ May 2020 to 30^th^ April 2021). The start date of the study reflects when the first patients with COVID-19 were admitted to HJH and coincided with the onset of the first wave in South Africa. The study end date was chosen to represent a full 12 months of admissions, incorporating two full waves of infection, prior to the onset of the third wave.

**Participants:** all patients with laboratory-confirmed SARS-CoV-2 infection admitted from 1^st^ May 2020 until 30^th^ April 2021 to HJH and recorded on the National Institute for Communicable Diseases COVID-19 Hospital Surveillance (DATCOV) database were included in this study. All patients with a positive SARS-CoV-2 result were followed up from the day of admission until their final hospital outcome (discharge, transfer, or death). The follow-up period for in-hospital outcomes was determined by the length of each patients´ hospital stay, and ended at discharge, transfer, or death. Once patients had a laboratory-confirmed diagnosis of SARS-CoV-2, case notification forms were captured on DATCOV by the medical staff (doctors and nurses). The hospital´s infection prevention and control team followed up and entered clinical details and patient outcomes until patient discharge, transfer, or death to ensure completion during the hospital stay.

Initially, only symptomatic patients fulfilling screening criteria for COVID-19 were swabbed and tested, however as the pandemic progressed, all medical patients had SARS-CoV-2 polymerase chain reaction (PCR) testing on admission, regardless of the reason for admission. Rapid antigen tests and serological assays were not available for diagnosis at this institution during the study period.

**Variables and data sources:** individual variables, diagnoses, and medical details were documented as reported in the clinical records by clinicians´ assessment, without pre-defined criteria. Data collected included demographic details (age, sex, and ethnicity), admission dates and details (general ward, high care, ICU), and patient outcomes (discharged, transferred, death). As this is an in-patient study, the outcomes include discharge, transfer, and death. “Discharge” was defined as a patient sent back from the hospital alive to their place of residence or rehabilitation facility after admission. “Transfer” was defined as a patient sent from this hospital to another health care facility or hospital for ongoing patient care. “Death” was defined as a patient death that occurred after the diagnosis of COVID-19 during the same hospital admission. These are described as COVID-19-associated deaths as individual causes of death were not confirmed with post-mortem data and death certificates were not analyzed as part of this study. Clinical details included oxygen and ventilation requirements, baseline conditions (obesity and smoking status), and comorbid illnesses, including hypertension, DM, cardiac disease, renal disease, malignancy, HIV, TB, chronic obstructive pulmonary disease (COPD), and asthma.

**Study size:** all patients admitted with laboratory-confirmed SARS-CoV-2 infection at HJH over 12 months were included in this study, thus sample size calculations were not used. The final number of patients analyzed was 1359.

**Bias:** to address potential selection bias, all patients with COVID-19 admitted to HJH during the study period with laboratory-confirmed SARS-CoV-2 infection were included in the analysis. According to national and institutional protocols at the beginning of the COVID-19 pandemic, only symptomatic patients with typical respiratory symptoms fulfilling the suspected COVID-19 case definition were tested, thus some patients with asymptomatic or atypical symptoms may have been missed during the first wave. However, as the pandemic progressed, all medical patients had SARS-CoV-2 PCR testing on admission. To address potential information bias, data were collected as documented by clinicians treating the patients, and these clinicians were not involved in the data analysis. The statistical analysis was conducted on anonymized data by an independent individual, not involved in patient care.

**Quantitative variables:** quantitative variables included age, (derived from the date of birth) and length of hospital admission, which was calculated from the dates of admission to outcome (discharge, death, or transfer). Age bands analyzed were as follows: less than 40 years, 41-60 years, 61-80 years, and greater than 80 years. This enabled comparison to other studies, which had demonstrated a significantly increased risk of mortality with increasing age, with an inflection point at 60 - 65 years of age [7-9].

The data was analyzed by waves (defined nationally as the period in which the weekly incidence risk of confirmed cases increased above 30 per 100,000 individuals [2]) of infections. In this study, the first wave is defined as 1^st^ May 2020 to 17^th^ October 2020 and was dominated by the ancestral D614G strain, and the second wave 18^th^ October 2020 to 30^th^ April 2021, dominated by the Beta variant. Hospital admissions and deaths due to COVID-19 are often delayed and occur in the period after the peak, and in South Africa, these admissions were usually followed a week or two after the increase in new cases reported with each wave [12,17]. In this study, the second wave was taken as starting halfway through the inter-wave period (between the peaks of each wave), to incorporate the delay in mortality following infection and admission from the first wave [3].

**Statistical methods:** categorical data were summarized as frequencies and percentages while continuous data were summarized as mean and standard deviation (SD) if normally distributed or median (interquartile range (IQR)) if otherwise. The normality assumption was assessed using the Shapiro-Wilk test. Bivariate tests were performed to allow between-wave comparisons. The Chi-square test was used to compare categorical variables between waves, an independent T-test was used to compare mean differences between waves, and the Mann-Whitney test was used to compare median differences between waves. The admission outcomes (discharged, death, transferred) were reported as frequencies and percentages. Risk factors for COVID-19-associated mortality in this setting were identified and quantified, by comparing these risk factors in survivors to non-survivors. Univariate and multiple logistic regression was used to determine the risk factors of COVID-19-related mortality. Stepwise variable selection was performed to select variables into the multiple logistic regression model. Variables in the adjusted logistic regression model included the wave period, age, sex, ethnicity, oxygen required, ventilation required, length of stay, hypertension, diabetes, cardiac disease, and people living with HIV. Variable were selected for significance, and excluded at a p-value of 10%, other than hypertension, DM, and HIV which were included as the most common co-morbidities. Overall, significance was set at 5% and the analysis was conducted using Stata version 17.

**Ethical considerations:** this research was approved by the Faculty of Health Sciences Human Research Ethics Committee (Medical) of the University of the Witwatersrand (M210665).

## Results

**Figure 1:**
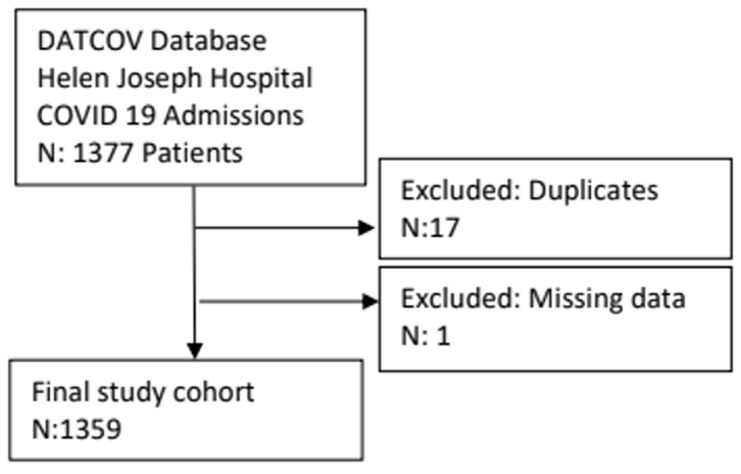
study population selection flow chart of COVID-19 admissions at Helen Joseph Hospital from 1^st^ May 2020 - 31^st^ April 2021

**Participants:** the database had 1377 COVID-19-infected patients during the study period. After editing for duplicates by date of birth, name, surname, and identity number, seventeen duplicate entries were deleted. One patient was removed from the database due to missing information. The final number of patients analyzed was 1359. For the study population selection flow chart of COVID-19 admissions is indicated as in Figure 1.

**Figure 2:**
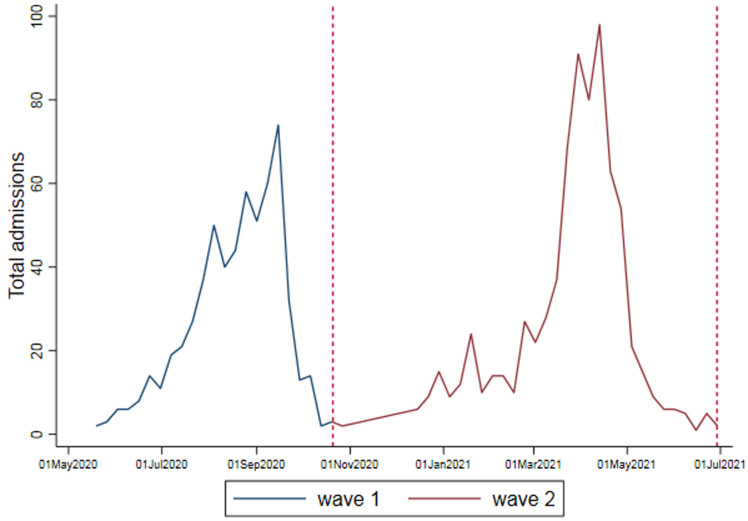
COVID-19 patient admissions per week at Helen Joseph Hospital; wave 1: 1^st^ May 2020 - 17^th^ October 2020; wave 2: 18^th^ October 2020 to 30^th^ April 2021

**Descriptive data:** of the 1359 patients, 595 (43.8%) were admitted during wave one, and 764 (56.2%) were admitted during wave two. Table 1 shows the demographic characteristics of the patients. Figure 2 shows the admissions per week over both waves. Overall, the mean (SD) age of patients admitted was 54.6 (15.4) years; 52.4% (n=711) were males, 64.0% of patients admitted were in the age groups 40-69 years, and the majority were of Black ethnicity (75.1%). There was no significant difference between the age (p: 0.414) and sex (p: 0.913) of patients admitted during the two waves.

**Table 1:** characteristics of COVID-19 patients admitted to Helen Joseph Hospital, 1^st^ May 2020 - 31^st^ April 2021, N=1359

Demographics	Total N=1359(%)	Wave 1 N=595 (%)	Wave 2 N=764 (%)	P-value
**Age**				
Mean (SD)	54.6 (15.4)	54.3 (15.3)	54.9 (15.5)	0.414
Sex				
Female	647 (47.6)	282 (47.5)	365 (47.8)	0.913
Male	711 (52.4)	312 (52.5)	399 (52.2)	
**Age groups**				
<30 years	71 (5.2)	27 (4.5)	44 (5.8)	
30-39 years	175 (12.9)	89 (15.0)	86 (11.3)	
40-49 years	264 (19.4)	107 (18.0)	157 (20.6)	0.321
50-59 years	299 (22.0)	139 (23.4)	160 (20.9)	
60-69 years	306 (22.5)	129 (21.7)	177 (23.2)	
70-79 years	181 (13.32	77 (12.9)	104 (13.6)	
80+ years	63 (4.6)	27 (4.5)	36 (4.7)	
**Ethnicity**				
Black	1021 (75.1)	406 (68.2)	615 (80.5)	
Mixed race	135 (9.9)	71 (11.9)	64 (8.4)	
Indian	31 (2.3)	23 (3.9)	8 (1.1)	
White	45 (3.3)	11 (1.9)	34 (4.5)	<0.001
Other	59 (4.3)	21 (3.5)	38 (5.0)	
Unknown	68 (5.0)	63 (10.6)	5 (0.7)	
**Outcome**				
Deaths	376 (27.7)	181 (30.4)	195 (25.5)	
Discharged	904 (66.5)	359 (60.3)	545 (71.3)	<0.001
Transferred out	79 (5.8)	55 (9.2)	24 (3.1)	
**Highest level of care**				
General ward	1263 (92.9)	575 (96.6)	688 (90.1)	<0.001
High care and ICU	96 (7.1)	20 (3.4)	76 (10.0)	
Ventilated				
No	1288 (4.8)	591 (99.3)	697 (91.2)	<0.001
Yes	71 (5.2)	4 (0.7)	67 (8.8)	
Requiring oxygen				
No	364 (26.8)	206 (34.6)	158 (20.7)	<0.001
Yes	995 (73.2)	389 (65.4)	606 (79.3)	
Length of stay in days				
Median (IQR)	8 (5-14)	7 (4-13)	9 (6-15)	<0.001

### Outcome data

**Hospital admission details and outcomes:** Table 1 shows the results of hospital admission and outcomes of the COVID-19-infected patients. The in-hospital CFR during the first wave was 30.4% (n=181/595), and 25.5% (n=195/764) in the second wave, and overall, 27.7% (n=376/1359) (p<0.001). Over both waves, 7.1% (96) patients were admitted to high care and the ICU, 73.2% (n=95) required oxygen, and 5.2% (n=71) were ventilated. In the first wave 65.4% (n=389) patients required oxygen while in hospital, with 79.3% (n=606) in the second wave. There were more high and intensive care patients in wave two (10.0%, n=76) than in wave one (3.4%, n=20), (p<0.001) and more patients were ventilated in wave two (8.8%, n=67) than wave one (0.7 %, n=4), (p<0.0001). The overall median length of stay in the hospital was 8 days (IQR 5-14), during the first wave the median was 7 days (IQR 4-13), and 9 days (IQR 6-15) in the second wave. Patients who were admitted in wave two stayed longer (9 days) compared to those who were admitted during wave one (7 days) (p<0.001).

**Baseline comorbidities and health conditions:** Table 2 shows the comorbid conditions of COVID-19-infected patients. Overall, the most common were hypertension (36.7%, n=499), DM (26.6%, n=362), HIV (10.8%, n=147), and obesity (11.0%, n=149). There was a high level of “unknown” status for all comorbidities, particularly HIV status, which was recorded as unknown for 51.4% (n=698) of all patients. These comorbidities were significantly different between wave one and wave two (p<0.05). Of the total number of patients living with DM (362), 82.5% (n=279) were known with pre-existing DM, and 17.5% (n=59) were newly diagnosed. Overall, 91.1% (n= 308) of patients with DM were documented as well controlled by treating clinicians, although this study did not assess hemoglobin a1C (HbA1C) levels. In the group of patients known to be living with HIV, 54.4% (n=80) were on antiretroviral treatment (ART), and 40.1% (n=59) had an unknown history regarding antiretroviral therapy (ART). At the time of data collection, 71.3% (n=57) of patients with HIV had an unknown HIV viral load, and only 22.5% (n=18) were known to be virally suppressed. The median clusters of differentiation 4 (CD4) count was 170 cells per microliter (n=36/147, IQR: 53.5 - 309).

**Table 2:** comorbid illnesses and conditions of COVID-19 patients admitted to Helen Joseph Hospital, 1^st^ May 2020 - 31^st^ April 2021, N=1359

Characteristics	Total n (%)	Wave 1 n (%)	Wave 2 n (%)	P-value
**Hypertension**				
No	397 (29.2)	155 (26.1)	242 (31.7)	
Yes	499 (36.7)	188 (31.7)	311 (40.7)	<0.001
Unknown	462 (34.0)	251 (42.3)	211 (27.6)	
**Diabetes mellitus (DM)**				
No	473 (34.8)	169 (28.4)	304 (39.8)	
Yes	362 (26.6)	167 (28.1)	362 (26.6)	<0.001
Unknown	524 (38.6)	259 (43.5)	524 (38.6)	
**Cardiac disease**				
No	464 (34.1)	201 (33.8)	263 (34.4)	
Yes	66 (4.9)	52 (8.7)	14 (1.8)	<0.001
Unknown	829 (61.0)	342 (57.5)	487 (63.7)	
**Chronic pulmonary disease**				
No	502 (36.9)	277 (38.2)	275 (36.0)	
Yes	8 (0.6)	7 (1.2)	1 (0.1)	0.028
Unknown	849 (62.5)	361 (60.7)	488 (63.9)	
**Asthma**				
No	551 (40.5)	239 (40.2)	312 (40.84	
Yes	41 (3.0)	17 (2.9)	24 (3.1)	0.914
Unknown	767 (56.4)	339 (57.0)	428 (56.0)	
**Chronic kidney disease**				
No	502 (36.9)	25 (37.8)	277 (36.3)	
Yes	34 (2.5)	25 (4.2)	478 (62.6)	0.001
Unknown	823 (60.6)	345 (58.0)	9 (1.2)	
**Malignancy**				
No	517 (38.0)	236 (39.7)	281 (36.8)	
Yes	2 (0.2)	1 (0.2)	1 (0.1)	0.542
Unknown	840 (61.8)	358 (60.2)	482 (63.1)	
**Obesity**				
No	473 (34.8)	173 (29.1)	300 (39.3)	
Yes	149 (11.0)	89 (15.0)	60 (7.9)	<0.001
Unknown	737 (54.2)	333 (56.0)	404 (52.2)	
**People living with HIV (PLWH)**				
No	514 (37.8)	189 (31.8)	325 (42.5)	
Yes	147 (10.8)	62 (10.4)	85 (11.1)	<0.001
Unknown	698 (51.4)	344 (57.8)	354 (46.3)	
**Active tuberculosis**				
No	563 (41.4)	236 (39.7)	327 (42.8)	
Yes	20 (1.5)	13 (2.2)	7 (0.9)	0.097
Unknown	776 (57.1)	346 (58.2)	430 (56.3)	
**Past tuberculosis**				
No	562 (41.4)	236 (39.7)	326 (42.7)	
Yes	13(1.0)	7 (1.2)	6 (0.8)	0.435
Unknown	784 (57.7)	352 (59.2)	432 (56.5)	
**Smoking status**				
Currently smoking	44 (3.2)	26 (4.4)	18 (2.4)	
Former smoker	65 (4.8)	29 (4.9)	36 (4.7)	<0.001
Never smoked	434 (31.9)	152 (25.6)	282 (36.9)	
Unknown	816 (60.0)	388 (65.2)	428 (56.0)	

### Main results

**Factors associated with in-hospital mortality:** the CFR was analyzed per variable, and univariate and multiple variable logistic regression results are shown in Table 3. Patients admitted in the first wave had a higher risk of dying compared to the second wave (adjusted odds ratio (aOR) 1.79; 95% confidence interval (CI) 1.35 - 2.38). Male patients had an increased risk of mortality (aOR 1.28; 95% CI 0.98 - 1.67). Non-Black African patients had a lower risk of mortality, with an adjusted odds ratio of 0.63 (CI 0.45- 0.88). Increasing age was associated with an increased risk of mortality, with the odds of mortality increasing by 4% (aOR 1.04; CI: 1.03-1.05) for each year increase in age. Patients requiring oxygen had 2.17 increased odds of mortality (aOR 2.17; 95% CI 1.56-3.01) and those requiring ventilation had 7.23 increased odds of mortality (aOR 7.23; 95% CI 4.02 - 13.01). Patients who stayed a day longer in the hospital had 3% reduced odds of death (aOR=0.97; 95% CI: 0.95-0.98).

**Table 3:** factors associated with in-hospital mortality of COVID-19 patients admitted to Helen Joseph Hospital, 1^st^ May 2020 - 31^st^ April 2021, N=1359

Characteristics	Case fatality ratio	Univariate logistic regression		Adjusted logistic regression	
	n/N (%)	Crude OR (95%CI)	p-value	Adjusted OR (95%CI)	p-value
**Wave period**					
1	181/595 (30.4)	1.28 (1.01- 1.62)	0.046	1.79 (1.35- 2.38)	<0.001
2	195/764 (25.5)	1(ref)		1(ref)	
**Age in years**					
Mean (SD)	60.9 (14.6)	1.04 (1.03-1.05)	<0.001	1.04 (1.03- 1.05)	<0.001
**Sex**					
Female	181/647 (28.0)	1(ref)		1(ref)	
Male	195/711 (27.4)	0.97 (0.77-1.23)	0.821	1.28 (0.98- 1.67)	0.072
**Age groups**					
<40 years	40/264 (15.2)	1(ref)			
41-60 years	126/579 (21.8)	1.56 (1.05-2.3)	0.026	-------	------
61-80 years	180/464 (38.8)	3.55 (2.41-5.21)	<0.001		
>80 years	30/52 (57.7)	7.64 (4.01-14.55)	<0.001		
**Ethnicity**					
Black African	292/1021(28.6)	1(ref)		1(ref)	
Non-black African^1^	69/270 (25.6)	0.84 (0.62-1.14)	0.266	0.63 (0.45- 0.88)	0.008
Unknown	16/68 (23.5)	0.77 (0.43-1.37)	0.37	0.57 (0.31-1.12)	0.096
**Smoking status**					
Never smoked	105/ 434(24.2)	1(ref)			
Ever smoked	35/109 (32.1)	1.48 (0.94-2.34)	0.092	------------	------
Unknown	236/ 816(28.9)	1.27 (0.97-1.66)	0.074		
**Level of care**					
General ward	346/1263 (27.4)	1(ref)			
High care /ICU	30/96 (31.3)	1.21 (0.77-1.89)	0.416	------------	-----
**Ventilation required**					
No	340/1288 (26.4)	1(ref)		1(ref)	
Yes	36/71 (50.7)	2.87 (1.77-4.64)	<0.001	7.23 (4.02- 13.01)	<0.001
**Oxygen required**					
No	67/364 (18.4)	1(ref)		1(ref)	
Yes	309/995 (31.1)	1.99 (1.48-2.69)	<0.001	2.17 (1.56- 3.01)	<0.001
**Length of stay (days)**					
Median (IQR)	5.5(3-11.5)	0.98 (0.97-0.99)	0.001	0.97 (0.95- 0.98)	<0.001
**Hypertension**					
No	81/397 (20.4)	1(ref)		1(ref)	
Yes	159/499 (31.9)	1.82 (1.34-2.48)	0.002	0.89 (0.58- 1.38)	0.735
Unknown	136/462 (29.4)	1.63 (1.18-2.23)	<0.001	0.91 (0.53 -1.57)	0.615
**DM**					
No	100/473 (21.1)	1(ref)		1(ref)	
Yes	114/362 (31.5)	1.71 (1.25-2.34)	<0.001	1.16 (0.76-1.77)	0.503
Unknown	162/524 (30.9)	1.67 (1.25-2.23)	0.001	1.37 (0.82- 2.28)	0.23
**Cardiac disease**					
No	96/464 (20.7)	1(ref)		1(ref)	
Yes	23/66 (34.8)	2.05 (1.17-3.57)	<0.001	1.37 (0.72- 2.64)	0.340
Unknown	257/829 (31.0)	1.72 (1.32-2.25)	0.011	1.76 (1.12 -2.76)	0.015
**Obesity**					
No	109/473 (23.0)	1(ref)			
Yes	50/149 (33.6)	1.69 (1.13-2.52)	0.015	------------	------
Unknown	217/737 (29.4)	1.39 (1.07-1.82)	0.011		
People living with HIV					
No	126/514 (24.5)	1(ref)		1(ref)	
Yes	31/147 (21.1)	0.82 (0.53-1.28)	0.009	0.94 (0.57 -1.58)	0.825
Unknown	219/698 (31.4)	1.41 (1.08-1.82)	0.39	0.88 (0.57- 1.36)	0.579
**Any infectious disease^2^**					
No	141/603 (23.4)	1(ref)			
Yes	20/93 (21.5)	0.89 (0.53-1.52)	0.689	------------	------
Unknown	215/663 (32.4)	1.57 (1.23-2.02)	<0.001		
**Any non-communicable^3^**					
No	149/636 (23.4)	1(ref)			
Yes	117/361 (32.4)	1.57 (1.18-2.09)	0.002		
Unknown	110/362 (30.4)	1.43 (1.06-1.91)	0.016	-------	
**Number of comorbidities**					
None	155/605 (25.6)	1(ref)			
One	110/404 (27.2)	1.09 (0.82-1.45)	0.570		
Two or more	111/350 (31.7)	1.35 (1.01-1.81)	0.043	------------	------

____: variable not included in the adjusted model; ^1^: non-black African: Coloured, White, Indian, and other; ^2^: underlying infectious disease: HIV, TB (both active and past TB); ^3^: non-communicable diseases and conditions: hypertension, DM, cardiac disease, chronic pulmonary disease, asthma, chronic kidney disease, malignancy, obesity; DM: diabetes mellitus

## Discussion

**Key results:** this study showed an overall in-hospital CFR of 27.7%, with 30.4% in wave one and 25.5% in wave two. This was at the higher end of COVID-19 associated in-hospital mortality rates in South Africa at that time, which ranged from 17-40% [11,12,15]. The high mortality rate in this study may be due to the strain on hospital capacity, which results in increased mortality during periods of greater volumes of hospital admissions, and the impact of different lineages of SARS-CoV-2 [12]. Beta lineage predominated during wave two in South Africa, although in this study, genomic sequencing and comparison of variant/non-variant cases was not performed [18]. Increased disease severity may also be expected in this facility, as it is a tertiary hospital, which admits patients from referring institutions and those with more complex disease.

These results are in contrast with national data, both the public and private sector, which showed increased mortality in wave two compared to wave one [12]. The decrease in mortality rates from wave one to wave two in this study may primarily be explained by the change in testing strategy during the study period at this facility, from initially limiting SARS-CoV-2 testing to patients with typical respiratory symptoms as identified on the screening questionnaire, to later testing all admissions, regardless of the indication for admission. This change in testing strategy likely identified more asymptomatic and minimally symptomatic patients with COVID-19 in wave two, who would be at lower risk for mortality. Other factors that may have contributed to the decrease in mortality in the second wave include changes in clinical guidelines and practice such as the use of steroids (after the Randomised Evaluation of COVID-19 Therapy (RECOVERY) trial data was released in June 2020), changes in ventilation strategies and increased hospital capacity and ventilatory equipment [19,20]. However, this decrease in mortality in the second wave was not reflected in national data, thus it is more likely to represent changes in the testing strategy and greater treatment capacity at this institution.

Demographically this cohort reflected both national and global trends, with the mean age of patients at 54.6 years, male patients accounting for 52.4% of admissions and over 60% of patients admitted were in the age groups 40-69 years. The low numbers of patients admitted to ICU/high care during the first wave, increasing in the second wave, while transfers out dropped, reflects the expansion in the hospital´s high care and ICU care.

This study was done prior to the availability of COVID-19 vaccines to the general public in South Africa which may have contributed to the high mortality rate described. Although vaccinations were available in South Africa from February 2021, this was limited to health care workers in a research trial during phase 1 of the national COVID-19 vaccine rollout strategy. This expanded to the public in phase 2, from May 2021, thus all patients included in this study were not vaccinated, as the study included patients admitted from 1^st^ May 2020 to 30^st^ April 2021 and did not involve health care workers.

In keeping with international data, significant risk factors for mortality included increasing age, as well as oxygen and ventilatory needs. Patients requiring oxygen had approximately double the risk of death, and those requiring ventilatory support had seven times the risk of in-hospital mortality. This likely represents patients admitted with more severe COVID-19 disease, who became critically ill, and therefore had a higher risk of mortality. It is known that COVID-19 patients who require high care and ICU admission have higher illness severity scores and a greater likelihood of multiorgan disease, which is associated with increased mortality [14,21]. In keeping with other South African data, non-Black African patients had a lower risk of mortality due to COVID-19 [11]. In this study, ward location was not associated with mortality, but this may be explained by the low numbers of patients admitted to high care and ICU.

In this study, both communicable and non-communicable diseases did not demonstrate increased risk of mortality with statistical significance, likely due to the relatively small sample size, and the high number of patients with missing information regarding comorbidities. Co-infection with HIV has been shown to increase the risk of mortality due to COVID-19 by 20% in a global meta-analysis and to double the risk in large South African study [22,23]. This analysis did not identify TB and HIV as risk factors for mortality, although this may be explained by the low numbers of patients known with these conditions in this cohort.

**Limitations:** this study had some limitations. These include the high proportion of “unknown”/missing data, especially the comorbidities, which may have impacted on strength of the associations found or biased estimates of the findings. Data collected on admission (usually when COVID-19 diagnosis was made), may be based on patient history rather than investigations (due to delayed laboratory or other results) and may therefore have under-evaluated the patient´s comorbid conditions and their extent, severity, or control. Due to severe or critical illness, patients might not have responded or provided a complete history. No genomic sequencing was done and comparison of variant/non-variant cases was not performed for the patients included in this study. However, genomic sequencing nationally has revealed that the wave periods were distinctly characterised by specific circulating viral variants described above.

This study has analysed the data in two waves, based on the date of hospital admission and according to a simplified case incidence into the first and second wave. These waves were determined based on peak national rates of new infections rather than a mathematical model or local time series analysis to determine the trajectory of the waves. Analysis by national waves of new infections may not account for the provincial heterogeneity in the timing of these waves. However, as Figure 1 shows, this division into two waves still reflects two clear admission peaks during this period.

This study did not analyse individual causes of death and was therefore does not distinguish deaths directly due to COVID-19 from deaths due to another cause in patients with incidental SARS-CoV-2 infection. However, the number of patients requiring oxygen (65.4% in the first wave and 79.3% in the second wave) indicated that a large proportion of patients had significant respiratory illness prior to their demise. COVID-19 infection was thus the likely cause of death in the majority of these patients. In addition, the hospital had postponed all elective procedures and surgery, thus hospital admissions at this time were predominantly medical admissions and emergency cases.

Finally, this analysis included all patients captured in the DATCOV system; it is possible that some patients were not captured, and some information might not have been captured thoroughly by the data capturers.

## Conclusion

This study compared the socio-demographic characteristics, clinical characteristics and hospital admission outcomes between COVID-19 wave one and wave two; and determined the factors associated with mortality among patients admitted with COVID-19 at HJH, Johannesburg. It demonstrated an overall in-hospital CFR of 27.7%. This high mortality rate represents the impact of the pandemic in the public sector with limited resources and capacity for ICU care, prior to the availability of COVID-19 vaccines. Risk factors for mortality included increasing age, male sex, and oxygenation and ventilation requirements. Understanding risk factors for COVID-19 associated mortality during the initial waves of the pandemic allows for risk stratification and prioritization of patients for preventive measures including vaccinations in future waves.

### What is known about this topic

- *COVID-19 inpatient mortality was high in the first waves of the pandemic, prior to the availability of SARS-CoV-2 vaccines;*
- *The first two waves were dominated in South Africa by the ancestral D614G strain and the Beta variant, which were associated with higher mortality than later variants, including Omicron;*
- *Risk factors for inpatient mortality due to COVID-19 include demographic factors, co-morbid illnesses (primarily non-communicable diseases), multiorgan involvement, and increased disease severity*.

### What this study adds

- *This year-long study during the first two waves of the pandemic gives a detailed analysis of the socio-demographic characteristics, clinical characteristics, and outcomes of patients admitted with COVID-19 at a tertiary public hospital in Johannesburg;*
- *This study shows the impact of the pandemic on the public sector in South Africa with limited resources and minimal capacity for high care and intensive care;*
- *Understanding risk factors for mortality due to COVID-19 enables risk stratification and prioritization of patients for preventative measures such as vaccinations in future waves*.
